# Oral administration of irinotecan in patients with solid tumors: an open-label, phase I, dose escalating study evaluating safety, tolerability and pharmacokinetics

**DOI:** 10.1007/s00280-018-3720-7

**Published:** 2018-11-08

**Authors:** I. Kümler, P. Grundtvig Sørensen, J. Palshof, E. Høgdall, W. Skovrider-Ruminski, S. Theile, A. Fullerton, P. G. Nielsen, B. Vittrup Jensen, D. L. Nielsen

**Affiliations:** 1 Department of Oncology, Herlev and Gentofte Hospital, Herlev, Denmark; 2 Oncoral Pharma ApS, c/o Jusmedico, Kongevejen 371, Holte, Denmark; 3 Department of Pathology, Herlev and Gentofte Hospital, Herlev, Denmark

**Keywords:** Phase I, Oral irinotecan, Dose finding

## Abstract

**Background:**

Oral drug formulations have several advantages compared to intravenous formulation. Apart from patient convenience and favorable pharmacoeconomics, they offer the possibility of frequent drug administration at home. In this study, we present a new oral irinotecan formulation designed as an enteric coated immediate release tablet which in pre-clinical studies has shown good exposure with low variability.

**Methods:**

A phase I, dose escalating study to assess safety, tolerability, pharmacokinetics and efficacy of an oral irinotecan formulation and to establish the maximum tolerated dose (MTD). Each treatment cycle was once-daily irinotecan for 14 days followed by 1 week rest.

**Results:**

25 patients were included across four cohorts; 3 patients were included in cohort 1 (20 mg/m^2^), 7 patients were included in cohort 2 (30 mg/m^2^), 3 patients were included in cohort 3 (25 mg/m^2^) and 12 patients were included in cohort 4 (21 mg/m^2^). Median age was 67 years, 52% were performance status (PS) 0 while 48% were PS 1. Median number of prior therapies was 3 (range 1–6). MTD was established at 21 mg/m^2^. No responses were observed. Nine patients (36%) had stable disease (SD), lasting median 19 weeks (range 7–45 weeks). Among these five patients had previously received irinotecan. No grade 3/4 hematologic toxicities were reported. Totally six patients experienced grade 1/2 anemia, three patients had grade 1/2 leucopenia and 1 patient had grade 1 thrombocytopenia. Most common non-hematological grade 1 and 2 adverse events were nausea, fatigue, diarrhea, vomiting and cholinergic syndrome. Grade 3 toxicities included diarrhea, fatigue, nausea and vomiting, no grade 4 events were reported. PK data showed consistent daily exposures during treatment at days 1 and 14 and no drug accumulation. SN-38 interpatient variability was in the same range as after infusion.

**Conclusions:**

Oral irinotecan was generally well tolerated; side effects were manageable and similar in type to those observed with intravenous irinotecan. Hematological toxicities were few and only grade 1/2. In this heavily pre-treated patient population, oral irinotecan demonstrated activity even among patients previously treated with irinotecan.

## Introduction

Irinotecan has been used in the treatment of various solid tumors for the past 2 decades and constitutes a corner stone in the treatment of metastatic colorectal, pancreatic and gastric cancers [1–7].

Originally introduced as an intravenous therapy, the search for an oral formulation has been ongoing almost since the drug was introduced [8]. Oral drug formulations are generally preferred by patients due to comfort and convenience, they often carry health economic benefits and, in the case of irinotecan, some studies indicate that oral irinotecan might be more effectively converted to the active metabolite SN-38 compared to intravenous administration [9, 10]. Further, oral administration allows for the concept of frequent dosing i.e., giving the chemotherapy at frequent time intervals in lower doses.

Irinotecan is a pro-drug which is enzymatically converted to the biologically active metabolite SN-38 [11]. Whereas irinotecan is water soluble, SN-38 is practically insoluble in water. Following metabolization in the liver SN-38 is converted to the water-soluble but inactive metabolite SN-38G. SN-38 is 100–1000 times more potent than irinotecan but the fraction of irinotecan actually converted to active SN-38 is small and exhibits a very large inter-patient variability. Due to severe side effects this problem is not readily solved by increasing the dose of irinotecan [12, 13]. During the past 20 years, several studies including various oral formulations of irinotecan have been conducted [8, 9, 14–19]. Oral bioavailability for both irinotecan and SN-38 has differed substantially among patients in the various studies [14–18]. The most common dose limiting toxicities have been diarrhea as well as nausea and vomiting. Despite fair efficacy results, none of the previously tested oral irinotecan formulations have gone into phase II trials mainly because of problems concerning poor solubility and a substantial interpatient variability in the oral bioavailability. In clinical trials, various oral formulations have been tested including solutions of i.v. irinotecan mixed with grape juice, powder filled capsules and semisolid matrix capsules, respectively. These formulations were all based on the water-soluble irinotecan hydrochloride, trihydrate salt [8–10, 18, 20]. More recently, preclinical studies have focused on improving solubility and increasing the bioavailability. In one study, SN-38 was encapsulated in lipid nano capsules and showed high permeability, however, only 8% of SN-38 was released after 3 days [21]. Another study investigated the conjugation of SN-38 to poly-amido amine dendrimers which resulted in increased trans-epithelial transport. Drug release was 10%, 20% and 56%, respectively, in simulated gastric, intestinal and liver environments [22]. Finally, pH-sensitive polymeric micelles of SN38 have been developed to improve permeability [23].

In the current study another approach has been used. The oral formulation in the present study is based on irinotecan in the free base form being solubilized in a hydrophobic lipid system. The system is formulated into an enteric coated tablet to avoid release in the stomach, as the stomach pH may influence the bio-absorption of irinotecan. The tablet releases the irinotecan immediately in the duodenum thereby avoiding protracted release. This is to ensure that the dosed irinotecan is eliminated before the next dose to avoid drug accumulation and to ensure high bio-absorption with low variability. In a pre-clinical repeat dose toxicity study, the irinotecan tablet was administered once daily for 14 days within a 3-week cycle and compared to irinotecan i.v. administration on day 1. The small daily doses of the irinotecan tablet resulted in less gastro-intestinal side effects compared to i.v. administration. Further, the white blood cell counts were less affected.

The aim of the present study was to evaluate a new oral tablet formulation of irinotecan in a clinical phase I study. The main purpose of the present study was to evaluate the safety and tolerability of the new oral formulation and to identify the maximal tolerated dose (MTD) in patients upon repeated oral dosing. Further, the pharmacokinetics including the interpatient variability were evaluated.

The study was designed as a standard dose escalating study with a subsequent extension trial in which the oral formulation was tested in combination with oral capecitabine. Only the first part of the study is described here.

## Patients and methods

### Study design

The study was a phase I, dose escalating, single center study to investigate the safety, tolerability and MTD. The investigational drug was designed and supplied by Oncoral Pharma ApS Denmark as an enteric coated immediate release tablet. The study drug was administered as tablets of either 7.5 mg or 10 mg. The total dose administered in mg was calculated as the body surface area (BSA) × dose in mg/m^2^. The number of tablets dispensed of each strength was selected to fit the total dose best possible.

One treatment cycle was 21 days and consisted of once-daily oral irinotecan for 14 days followed by 1 week rest, mimicking the most common used regimes with capecitabine.

The study was designed as a standard “3 + 3” design with an estimated 15–24 patients needed to establish the MTD. After reaching the MTD, an additional 12 patients were included to obtain sufficient data on safety and pharmacokinetics at the MTD level.

The starting dose was 20 mg/m^2^ daily followed by fixed increments of 10 mg/m^2^ with the opportunity to increase by only 5 mg/m^2^ in case of safety concerns. Progression to the next dose level was allowed if no dose limiting toxicities (DLT) were found in three patients. In case of a DLT occurrence, an additional three patients were to be included in the dose level and progression was only allowed if ≤ 1 patient experienced a DLT. If DLTs were found in ≥ 2 patients the study design was to be modified following safety data review. A safety board was established, and meetings were held prior to every dose escalation and on demand.

During the DLT period, i.e., the first two cycles, clinical safety assessment was performed at screening, on days 2, 5, 9, 14, 15, 22 in cycle one and once weekly in cycle two. Laboratory safety assessment was performed at screening, on days 5, 15 and 19 in cycle one and on days 8, 15 and 19 in cycle two.

DLT was defined as neutropenia or thrombocytopenia grade 4 or bleeding due to thrombocytopenia, any grade 3–4 adverse events thought to be treatment related, grade ≥ 3 diarrhea, vomiting or nausea despite optimal treatment, moderate to severe symptoms of early cholinergic syndrome or other adverse reactions leading to treatment delay for more than 2 weeks.

Pharmacokinetics of irinotecan, SN-38 and SN-38G were investigated on days 1 and 14 of the first cycle, 10 min pre-dosing and at 1 h, 1.5 h, 2 h, 2.5 h, 3 h, 3.5 h, 4 h, 5 h, 6 h, 8 h, 12 h and 24 h post dosing.

There were no restrictions on food intake during treatment except for days 1 and 14 of pharmacokinetic sampling where the patients were fasted from 10 p.m. in the evening and until 1 h post dosing.

Any impact of food was investigated at the dose level 21 mg/m^2^ (MTD) for six subjects taking the dose in fasted state on day 1 and in fed state on day 14, respectively.

Tumor response was evaluated according to RECIST 1.1 [24] with CT scans at day 36 (after 2 cycles of treatment) and hereafter every 6 weeks.

### UGT1A1

SN-38 is primarily metabolized to the inactive SN-38 glucuronide by uridine diphosphate-glucuronyl transferase 1A1 (UGT1A1). UGT1A1 is an enzyme responsible for catalyzing the glucuronidation of various compounds, including steroid hormones, bilirubin, as well as xenobiotics, such as irinotecan. A polymorphic variation in the promoter of UGT1A1 leads to decreased expression of UGT1A1, resulting in reduced glucuronidation of SN-38, the active metabolite of irinotecan. Some studies have indicated that the risk of irinotecan toxicity is increased among persons with genetic variants associated with reduced UGT enzyme activity. Thus, patients with a (TA)7 repeat (UGT1A1*28) are at increased risk of developing grade 4 neutropenia or severe diarrhea [25]. The FDA-approved drug label for irinotecan states that when irinotecan is administered as a single agent, a reduction in the starting dose by at least one level of irinotecan hydrochloride injection should be considered for patients known to be homozygous for the UGT1A1*28 allele [26, 27]. As a consequence, we decided to study the polymorphisms of UGT1A1 in all patients entering the study.

A UGT1A1 Genotyping Kit (UGT-RT50, EntroGen) was used. This is a polymerase chain reaction (PCR)-based assay with allele-specific probes, which identify the most common irinotecan polymorphic variant. Briefly, genomic DNA was extracted from whole blood (*n* = 25) and used for the multiplexed amplification of the promoter region of the UGT1A1 gene with oligonucleotide primers that flanks the UGT1A1*1/*28 SNP. The variants were detected using fluorescent dyes [FAM labeled probes detect UGT1A1*1 (wild type), VIC labeled probes detect UGT1A1*28 (mutant)]. Samples were classified as: homozygote (UGT1A1*1 or UGT1A1*28) and heterozygote (UGT1A1*1 and UGT1A1*28). Amplification was performed on an Applied Biosystems 7500 instrument.

### Study population

Patients with metastatic or unresectable solid tumors for whom no standard treatment options existed were eligible for inclusion. Patients were required to be in performance status 0–1 according to ECOG [28], have a life expectancy of ≥ 3 month and adequate organ and bone marrow function. Furthermore, patients with chronic enteropathy, bowel obstruction or sub-obstruction, prior history of malabsorption or symptomatic brain metastases were excluded.

### Study objectives

The primary objectives were to determine the safety, tolerability and MTD of oral irinotecan. Secondary objectives included pharmacokinetics and tumor response.

### Statistical analysis

No formal statistical analyses were performed on safety or efficacy data. Descriptive statistics were used for patient demographics, safety and efficacy data.

### Pharmacokinetic analysis

Concentrations of irinotecan, SN-38 and SN-38G in stabilized human plasma samples were measured using LC-MS/MS with protein precipitation extraction. The corrected lower limit of quantification (LLOQ) for irinotecan and SN-38 in human plasma was 0.110 ng/mL and 0.550 ng/mL for SN-38G. Values below were reported as below limit of quantification (BLQ). The extent of conversion of irinotecan to SN-38 was calculated as the metabolic ratio being AUC_SN-38_/AUC_Iri_. The extent of glucuronidation of SN-38 to SN-38G was calculated as the glucuronidation ratio being AUC_SN-38G_/AUC_SN-38_.

Pharmacokinetic non-compartmental analysis was performed using WinNonlin v6.3 on irinotecan and its metabolites SN-38 and SN-38G. A value of 0 was used for all human plasma concentrations recorded as BLQ prior to *t*_max_ and concentrations recorded as BLQ after *t*_max_ were set to missing. Estimation of *t*_½_ was subject to a minimum of 3 data points on the fitted line of regression, a measured portion of the line of regression equivalent to at least 1.5 times the half-life and a coefficient of determination (*R*^2^) of at least 0.80.

### Ethics

The study was performed in accordance with the Declaration of Helsinki, ICH-Good Clinical Practice and approved by the Regional Ethics Committee (H-15000878) of Denmark. All included patients provided written, informed consent. The study was initiated by the principal investigator at Herlev and Gentofte Hospital, Department of Oncology and partly sponsored by grants from the Innovation Foundation and the Danish Cancer Society and registered at EudraCT (2014-005584-32) and at ClinicalTrials.Gov (NCT03295084).

## Results

### Patient characteristics

From July 2015 to September 2017, 25 patients were included across 4 treatment cohorts; 3 patients were included in cohort 1 (20 mg/m^2^), 7 patients were included in cohort 2 (30 mg/m^2^), 3 patients were included in cohort 3 (25 mg/m^2^) and 12 patients were included in cohort 4 (21 mg/m^2^). According to the protocol, one patient in cohort 2 was replaced as this patient was withdrawn due to cancer-related complaints after only 4 days of dosing with irinotecan.

Included patients were median 67 years old (range 51–82), 52% were performance status (PS) 0 while 48% were PS 1. Among the included patients, six had a diagnosis of cholangiocarcinoma, five had a diagnosis of colon cancer, four had pancreas and prostate cancer, respectively, and one each had cervical cancer, NSCLC, ovarian cancer, rectal cancer, SCLC and mesothelioma. Median number of prior therapies was 3 ranging from 1 to 6.

Patients’ characteristics are summarized in Table 1.

**Table 1:** Patient demographics and disease characteristics (25 patients)

Characteristics	Number of patients (%)
Median age, years (range)	67 (51–82)
Gender	
Male	14 (56)
Female	11 (44)
ECOG performance status	
0	13 (52)
1	12 (48)
Primary cancer	
Cholangiocarcinoma	6 (24)
Colon	5 (20)
Pancreas	4 (16)
Prostate	4 (16)
Cervix	1 (4)
Mesothelioma	1 (4)
NSCLC	1 (4)
Ovarian	1 (4)
Rectum	1 (4)
SCLC	1 (4)
Extent of disease	
Locally advanced	5 (20)
Metastatic	20 (80)
Median number of prior regimen for advanced disease (range)	3 (1–6)
Prior treatment with irinotecan	
Yes	15 (60)
No	10 (40)
UGT1A1	
UGT1A1*1/1 homozygote	13 (52)
UGT1A1*1/28 heterozygote	10 (40)
UGT1A1*28/28 homozygote	2 (8)

### Dose escalation and dose limiting toxicities

In cohort 1, no DLTs were observed. At progression to next dose level (30 mg/m^2^), one patient experienced diarrhea grade 3 and thus three more patients were included. As one more patient experienced grade 3 diarrhea, the MTD was exceeded and the dose level was lowered to 25 mg/m^2^. Three patients were included; one grade 3 diarrhea accompanied with dehydration grade 3 was reported as well as elevated alkaline phosphatase grade 3.

Following a safety board meeting, the dose was lowered to 21 mg/m^2^ and this dose was accepted as MTD.

The median treatment duration in cohort 1–4 was 70 days (range 35–97), 9 days (range 4–35), 11 days (range 5–119) and 35.5 days (range 2–228), respectively. Total doses of irinotecan were 1640 mg, 450 mg, 440 mg and 1100 mg, respectively, in cohorts 1, 2, 3 and 4. The shorter treatment periods in cohorts 2 and 3 are reflected in the lower total doses. Among the 15 patients treated in either cohort 1 or 4 at the MTD, the median dose of irinotecan was 1120 mg (range 71–8190 mg).

### Pharmacokinetics

The pharmacokinetic parameters of Irinotecan, SN-38 and SN-38G were determined in totally 25 patients on day 1 and day 14 of the first cycle. As the three patients in cohort 1 (20 mg/m^2^) were dosed 20.9 mg/m^2^ and within the range of dosing for cohort 4 (21 mg/m^2^) the patients of cohorts 1 and 4 were merged into a common cohort 4, i.e., dose level 21 mg/m^2^ for the pharmacokinetic evaluation.

**Fig. 1:**
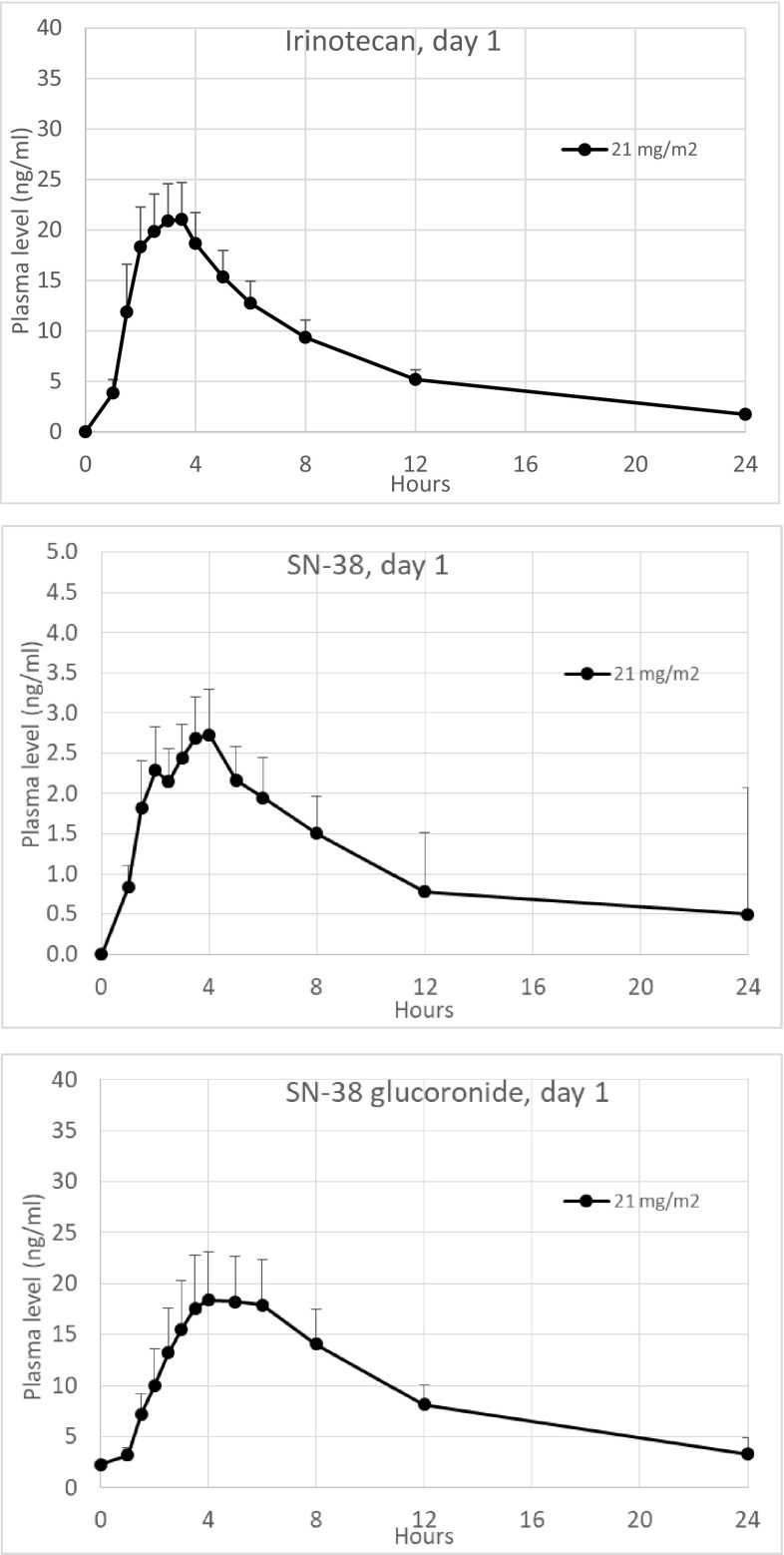
Pharmacokinetic plasma profiles of irinotecan, SN-38 and SN-38G on day 1 (mean values + SEM, *n* = 15)

The pharmacokinetic data for irinotecan, SN-38 and SN-38G on both days are provided in Table 2 and plasma profiles of irinotecan, SN-38 and SN-38G on day 1 are provided in Fig. 1.

**Table 2:** Pharmacokinetic parameters of irinotecan, SN-38 and SN-38G

Dose (no. patients)	Parameters	Irinotecan		SN-38		SN-38G	
		Day 1	Day 14^a^	Day 1	Day 14^a^	Day 1	Day 14^a^
21 mg/m^2^/day (*n* = 15)	*C*_max_ (ng/mL)	19.2 (16.5)	15.2 (9.54)	2.67 (2.41)	2.19 (1.92)	13.8 (22.2)	13.0 (8.85)
	*t*_max_ (h)	3.0 (1.5–6.0)	3.0 (1.5–6.0)	4.0 (1.5–5.0)	4.0 (2.0–8.0)	5.0 (3.5–6.0)	5.5 (2.5–12)
	AUC_0–24_ (ng h/mL)	135 (114)	133 (66.9)	22.5 (14.3)	21.6 (9.22)	149 (210)	146 (117)
	AUC_0–∞_ (ng h/mL)	142 (130)^a^	150 (75.0)^c^	31.3 (17.9)	19.7 (4.86)^e^	154 (263)	172 (63.0)^g^
	*t*_½_ (h)	6.78 (0.96)^a^	7.63 (0.99)^c^	7.88 (2.16)^d^	11.0 (1.10)^e^	7.63 (1.30)^b^	8.68 (0.62)^g^
	*C*_max_ (_Day14/Day1_)	–	0.85 (0.82)	–	0.87 (1.48)	–	0.94 (0.73)
	AUC_0–24_ (_Day14/Day1_)	–	1.08 (0.84)		1.00 (0.65)		1.06 (0.70)
25 mg/m^2^/day (*n* = 3)	*C*_max_ (ng/mL)	46.7 (12.1)	34.6 (*n* = 1)	7.91 (6.39)	2.81 (*n* = 1)	27.9 (16.6)	11.6 (*n* = 1)
	*t*_max_ (h)	2.0 (2–2.5)	2.0 (*n* = 1)	2.5 (2.0–4.0)	3.5 (*n* = 1)	3.5 (2.5–5.0)	3.5 (*n* = 1)
	AUC_0–24_ (ng h/mL)	317 (167)	237 (*n* = 1)	51.7 (46.8)	22.9 (*n* = 1)	261 (279)	100 (*n* = 1)
	AUC_0−∞_ (ng h/mL)	264 (*n* = 2)	274 (*n* = 1)	51.8 (*n* = 1)	NC	279 (*n* = 1)	NC
	*t*_½_ (h)	6.59 (*n* = 2)	8.88 (*n* = 1)	7.13 (*n* = 1)	NC	5.56 (*n* = 1)	NC
30 mg/m^2^/day (*n* = 7)	*C*_max_ (ng/mL)	70 (35.9)	70.5 (*n* = 2)	8.99 (6.23)	6.04 (*n* = 2)	38.8 (25.2)	24.3 (*n* = 2)
	*t*_max_ (h)	2.5 (1.5–3.0)	2.0 (*n* = 2)	2.5 (1.5–6.0)	1.8 (1.5–2.0)^f^	3.0 (2.5–6.0)	3.5 (*n* = 2)
	AUC_0–24_ (ng h/mL)	445 (191)	442 (*n* = 2)	58.5 (35.5)	39.0 (6.51)^f^	378 (329)	242 (*n* = 2)
	AUC_0−∞_ (ng h/mL)	497 (240)	493 (*n* = 2)	61.9 (*n* = 1)	NC	439 (236)^h^	285 (*n* = 2)
	*t*_½_ (h)	7.46 (1.29)	7.31 (*n* = 2)	8.92 (*n* = 1)	NC	8.56 (0.87)^h^	8.37 (*n* = 2)

Data as geometric mean (SD) except for *t*_max_ as median (range). Individual PK parameters are listed if *n* < 3

*NC* not calculated

^a^*n* = 14; ^b^*n* = 12; ^c^*n* = 8; ^d^*n* = 7; ^e^*n* = 3; ^f^*n* = 2; ^g^*n* = 4; ^h^*n* = 5

At the MTD level (21 mg/m^2^ dose level), mean *C*_max_ levels of 19.2 ng/mL and 15.2 ng/mL and mean AUC_0–24_ levels of 135 h ng/mL and 133 h ng/mL, respectively, were found on day 1 and day 14 for irinotecan. Median time to observe maximum concentration (*t*_max_) for irinotecan was 3 h on both days. For SN-38 mean *C*_max_ levels of 2.67 ng/mL and 2.19 ng/mL and mean AUC_0–24_ levels of 22.5 and 21.6 ng/mL were found with a median *t*_max_ of 4 h on both day 1 and day 14. The metabolic ratios of SN-38/irinotecan at MTD were consistent after 14 days of repeat dosing with AUC_0–24_ ratios of 0.175 (17.5%) and 0.163 (16.3%), and *C*_max_ ratios of 0.142 and 0.145 on day 1 and day 14, respectively. The metabolic AUC_0–24_ ratios of SN-38G/SN-38 were 6.38 and 6.78 and *C*_max_ ratios were 5.45 and 5.91 on day 1 and day 14, respectively.

The geometric means day 14 over day 1 exposure ratio of irinotecan for *C*_max_ and AUC_0–24_ were 0.85 and 1.08, respectively, and 0.87 and 1.00 for SN-38. The geometric mean trough concentration *C*_24_ prior to dosing at day 14 was 1.95 ng/mL for irinotecan and 0.631 ng/mL for SN-38. Both the geometric mean day 14 over day 1 exposure ratio and the *C*_24_ level indicate no drug accumulation of relevance for irinotecan and SN-38.

The inter-patient coefficient of variation (CV%) in SN-38 exposure (based on AUC_0–*t*_) was 63.3% on day 1 and 42.6% day 14 for SN-38.

The geometric mean AUC_0–*t*_ obtained was 159 h ng/mL and 124 h ng/mL in fasted and fed state, respectively. The fed/fasted ratio of the geometric mean AUC_0–24_ for the six patients taking the irinotecan tablet in fasted state at MTD on day 1 and in fed state on day 14 was 0.776 for irinotecan and 0.791 for SN-38 with a range from 0.145 to 2.17 for irinotecan and 0.218 to 1.58 for SN-38 as some patients experienced somewhat higher AUC_0–24_ on day 1 in fasted state and others on day 14 after a meal.

Increasing the doses to 25 mg/m^2^ and to 30 mg/m^2^ resulted in increases in both geometric mean *C*_max_ and AUC_0–*t*_ of irinotecan. For SN-38 the exposure increased with increasing dose in a manner that was greater than dose proportional between the 21 and 25 mg/m^2^ dose levels, and proportional between the 25 and 30 mg/m^2^ doses. In general, there was not enough data at day 14 for the dose levels 25 mg/m^2^ and 30 mg/m^2^ to draw any firm pharmacokinetic conclusions.

### UGT1A1

In total, 25 blood samples from patients included were analyzed. We did perform all analyses in duplicate to ensure consistent results. The detected variants found were classified as homozygote UGT1A1*1; 13 (52%), homozygote UGT1A1*28; 2 (8%) and heterozygote 10 (40%). Of two patients homozygote for UGT1A1*28, one patient experienced a grade 3 diarrhea and withdrew the study due to toxicity. The following CT scan showed stable disease (SD). The other patient terminated the study early due to cancer-related complaints and only received oral irinotecan for 4 days. Among the remaining patients with grade 3 diarrhea one was heterozygote for UGT1A1*28 and three were homozygote for UGT1A1*1. Additionally, patients with SD were found among both UGT1A1*1 homozygote and UGT1A1*28 heterozygote.

### Safety

Only grade 1 and 2 hematologic toxicities were reported. Totally six patients across all cohorts experienced grade 1/2 anemia, three patients had grade 1/2 leucopenia and only one patient had grade 1 thrombocytopenia (Table 3).

**Table 3:** Treatment-emergent abnormal hematological laboratory values

	20 mg/m^2^ (*n* = 3)		30 mg/m^2^ (*n* = 7)		25 mg/m^2^ (*n* = 3)		21 mg/m^2^ (*n* = 12)		Total (*n* = 25)	
	Grade 1 *n* (%)	Grade 2 *n* (%)	Grade 1 *n* (%)	Grade 2 *n* (%)	Grade 1 *n* (%)	Grade 2 *n* (%)	Grade 1 *n* (%)	Grade 2 *n* (%)	Grade 1 *n* (%)	Grade 2 *n* (%)
Hemoglobin	1 (33.3)	1 (33.3)	1 (14.3)	2 (28.6)	0	0	1 (8.3)	0	3 (12)	3 (12)
WBC	1 (33.3)	1 (33.3)	0	0	0	0	0	1 (8.3)	1 (4)	2 (8)
ANC	0	0	0	0	0	0	0	0	0	0
Platelets	0	0	0	0	0	0	1 (8.3)	0	1 (4)	0

Grade 3 or 4 decreased values were not observed

*ANC* absolute neutrophil count, *WBC* white blood cell count

Most common non-hematological grade 1 and 2 adverse events were nausea, fatigue, diarrhea, vomiting and cholinergic syndrome (Table 4). Across cohorts 1–3 DLTs were observed in four patients and included diarrhea (three patients) and increased alkaline phosphatase (one patient). In cohort 4, totally four patients experienced grade 3 AEs probably or possible related, these included diarrhea, nausea, vomiting, spleen infarction, cataract, increased blood bilirubin, fatigue and deterioration of general condition (Table 4). Totally 13 patients (52%) discontinued the study due to unacceptable side effects. In cohort 4, five patients (42%) discontinued due to toxicities.

**Table 4:** Treatment-related adverse events at MTD level 21 mg/m^2^ (12 patients)

	Grade 1/2 *n* (%)	Grade 3^a^ *n* (%)	All grade *n* (%)
Fatigue	4 (33.3)	1 (8.3)	5 (41.7)
Weight loss	4 (33.3)	0	4 (33.3)
Constipation	0	0	0
Diarrhea	6 (50.0)	1 (8.3)	7 (58.3)
Nausea	7 (58.3)	1 (8.3)	8 (66.7)
Vomiting	6 (50.0)	1 (8.3)	7 (58.3)
Cholinergic syndrome	4 (33.3)	0	4 (33.3)
Mucositis	4 (33.3)	0	4 (33.3)
Dyspnea	1 (8.3)	0	1 (8.3)
Fever	1 (8.3)	0	1 (8.3)
Febrile neutropenia	0	0	0

Grade 4 adverse events were not observed

^a^In addition the following grade 3 adverse events were observed; increased blood bilirubin, cataract (worsening), infarction of spleen and deterioration of general condition

### Efficacy

No complete or partial responses were observed. Nine patients (36%) had SD at first response evaluation, lasting median 19 weeks (range 7–45 weeks). Among patients with SD three had a diagnosis of colon cancer, three had cholangiocarcinoma and one each had SCLC, mesothelioma and ovarian cancer. Among patients with SD, five had previously received intravenous irinotecan.

Totally eight patients were non-evaluable as they discontinued the study due to toxicities and no CT scan was performed at the end of treatment.

## Discussion

Oral drug formulations possess several advantages compared to intravenous formulations. Apart from patient convenience and favorable pharmacoeconomics, they offer the possibility of out-hospital drug administration at frequent intervals at lower doses. As no oral irinotecan formulations have been approved, clinical data on frequent dosing with irinotecan are sparse. However, data from the few published studies seem to indicate that frequent dosing is well tolerated and possibly more efficacious than more protracted administration schedules [29–31].

Several attempts have been made to develop a safe and efficient oral administration of irinotecan using a dosing regimen of either 5 or 14 days of treatment within 3 weeks treatment cycles [8–10, 18, 20].

These studies indicate that it is most likely the cumulative dose within a treatment cycle that defines DLT and thereby the MTD. Treatment once daily for 5 days within a 3-week cycle resulted in MTDs in the range 50–80 mg/m^2^ of irinotecan, hydrochloride, and trihydrate, whereas treatment once daily for 14 days resulted in MTDs in the range of 30–40 mg/m^2^ of irinotecan, hydrochloride, and trihydrate. In the present study, a MTD of 21 mg/m^2^ irinotecan (free base) corresponding to 24 mg/m^2^ of irinotecan, hydrochloride, trihydrate was administered daily providing a cumulative dose of approximately 340 mg/m^2^ within each 3-week treatment cycle. This cumulative dose corresponds well with the commonly used dose 340 mg/m^2^ provided as i.v. administration every 3 weeks [27].

The pharmacokinetic data of the present study showed that irinotecan was rapidly and well absorbed and converted to its active metabolite SN-38 upon oral administration. Oral irinotecan was more effectively converted to SN-38 compared to intravenous administration as conversion was approximately six- to sevenfold higher following oral administration. The metabolic ratio of SN38/irinotecan was in the range of 16.3–16.6% compared to 2.3% following i.v. administration. The gastro-intestinal tract contains high levels of the carboxylesterase enzyme most likely responsible for the additional conversion to SN-38. Plasma peaks of irinotecan and SN-38 occurred after approximately 3 h. The absorbed irinotecan was eliminated within 24 h with no evidence of accumulation upon repeated dosing. Thus, the current treatment regimen appeared to be safe in relation to drug accumulation and provided consistent daily exposures during treatment from day 1 to day 14 within each cycle.

The cumulative exposure of SN-38 within a 3-week cycle was in the same range as that obtained following a single i.v. administration of 340 mg/m^2^ (approximately 315 ng/mL versus 474 ng/mL) [27].

The inter-patient coefficient of variation in SN-38 exposure (AUC_0–24_) after i.v. dosing has been reported to be approximately 47–51% and is most likely linked to the complex pharmacokinetics of the drug. The CV% for the active metabolite SN-38 after oral administration of the irinotecan tablet was 43–63% which is comparable to that of i.v. treatment [26].

The impact of food on oral irinotecan was investigated in six patients. In accordance with previously published data, no significant effect on absorption of irinotecan was found following food intake [19].

Grades 3 and 4 late diarrhea appear in 16–22% of patients after i.v. administration of irinotecan [32]. SN-38G is considered responsible for the delayed diarrhea through biliary excretion and increased SN-38 exposure in the gut. Totally five patients experienced grade 3 diarrhea after oral dosing in the present study, respectively, three patients after exposure to 30 mg/m^2^, one patient after exposure to 25 mg/m^2^ and one patient at MTD after oral dosing in the present study. However, no correlation was found between grade 3 diarrhea and the levels of SN-38G or the SN-38G/SN-38 ratios found in the patients.

UGT1A1 plays a role in the glucuronidation of SN-38 and it has been speculated whether a screening for UGT1A1*28 polymorphism could identify patients with lower SN-38 glucuronidation rates and a greater susceptibility to irinotecan induced diarrhea [33]. Although a limited number of patients were included in this study no correlation between UGT1A1 variants and toxicity or response was identified. This agrees with findings from another study of oral irinotecan where no correlation was found between irinotecan associated toxicity and the UGT1A1*28 genotype [19].

Non-hematological side effects such as late diarrhea, nausea and vomiting are common following i.v. administration. Grades 3 and 4 diarrhea were reported in 30.6% of patients, grades 3 and 4 nausea in 16.8% of patients and grades 3 and 4 vomiting in 12.5% of patients after i.v. administration [19]. The non-hematological side effects following administration of the irinotecan tablet were comparable to those observed following i.v. formulations but less severe [4, 6, 7]. In our study, most non-hematological side effects were of grade 1 or 2. Only one subject (8.3%) experienced grade 3 diarrhea, vomiting or nausea, respectively at the MTD level.

Myelosuppression is a common toxicity following i.v. administration of irinotecan and possesses a serious threat to patients on chemotherapy. Thus, while hematologic side effects grades 3 and 4 are reported following i.v. administration of irinotecan in 10–31.4% of patients participating in clinical trials [27, 34–37], no grade 3 or 4 hematologic toxicities were reported in the present study, indicating that the oral administration may have a favorable hematologic profile compared to intravenous administration. This is most likely due to the administration of small daily oral doses in contrast to a large dose administered intravenously weekly or every third week [4, 6, 7].

No objective responses were seen in this heavily pretreated cohort, however, nine (36%) patients did obtain a clinical benefit with SD lasting median 19 weeks. Among these, five patients (56%) had previously been treated with intravenous irinotecan.

In conclusion, we found the oral formulation to be safe and efficacious with less hematologic side effects. Our tablet formulation is currently investigated in combination with capecitabine.
